# Lipocalin 2 (LCN2) is a promising target for cholangiocarcinoma treatment and bile LCN2 level is a potential cholangiocarcinoma diagnostic marker

**DOI:** 10.1038/srep36138

**Published:** 2016-10-26

**Authors:** Kun-Chun Chiang, Ta-Sen Yeh, Ren-Chin Wu, Jong-Hwei S. Pang, Chi-Tung Cheng, Shang-Yu Wang, Horng-Heng Juang, Chun-Nan Yeh

**Affiliations:** 1 General Surgery Department, Chang Gung Memorial Hospital, Chang Gung University, Keelung, Taiwan, R.O.C; 2 Director of Zebrafish center of Keelung Chang Gung Memorial Hospital, Taiwan, R.O.C; 3 General Surgery Department and Liver research center, Chang Gung Memorial Hospital, Chang Gung University, Kwei-Shan, Taoyuan, Taiwan, R.O.C; 4 Department of Pathology and Liver research center, Chang Gung Memorial Hospital, Chang Gung University, Kwei-Shan, Taoyuan, Taiwan, R.O.C; 5 Graduate Institute of Clinical Medical Sciences, College of Medicine, Chang Gung University, Kwei-Shan, Taoyuan, Taiwan, R.O.C; 6 Department of Anatomy, College of Medicine, Chang Gung University, Kwei-Shan Taoyuan, Taiwan, 333, R.O.C; 7 Department of Urology, Chang Gung Memorial Hospital, Kwei-Shan, Tao-Yuan, Taiwan, ROC

## Abstract

Cholangiocarcinoma (CCA) is a devastating disease due to resistance to traditional chemotherapies and radiotherapies. New therapeutic strategies against CCA are urgently needed. This study investigated the role of lipocalin-2 (*LCN2*) in human cholangiocarcinoma as a potential therapeutic target and diagnostic marker. So far, the role of *LCN2* in cancer is still controversial and studies regarding the role of LCN2 in CCA are limited. *LCN2* knockdown inhibited CCA cell growth *in vitro* and *in vivo* through induction of cell cycle arrest at G0/G1 phases and decreased metastatic potential due to repression of epithelial-mesenchymal transition (EMT). Overexpression of *LCN2* in CCA cells increased cell metastatic potential. We showed for the first time that the N-myc downstream regulated gene 1 (*NDRG1*) and *NDRG2*, known as tumor suppressor genes, are negatively regulated by LCN2 in CCA cells. LCN2 concentration in bile was higher in patients with CCA than that in patients with gallstones, with a cutoff value of 20.08 ng/ml making this a potential diagnostic marker. Higher *LCN2* expression was associated with worse survival in patients with CCA. *LCN2* is a promising target for CCA treatment and bile LCN2 level is a potential diagnostic marker for CCA.

Cholangiocarcinoma (CCA) is an epithelial malignancy arising from the bile ducts, and ranks as the second most common liver malignancy, after hepatocellular carcinoma. Recently, due to increased recognition and incidence, the interest in treatments for this cancer has increased^1^. Most CCA develops *de novo* without obvious risk factors. The 5 year survival rate of CCA is very low due to late diagnosis and resistance to traditional anti-cancer regimens^2^. Curative radical surgery remains the standard and most effective treatment for CCA; however, most patients with CCA are not good candidates for operation due to advanced disease at the time of diagnosis. Thus, the development of new therapeutic targets for CCA should be prioritized.

Lipocalin-2 (LCN2), also known as NGAL, uterocalin, or 24p3, belongs to the lipocalin superfamily. LCN2 is a secreted protein with the ability to interact with other ligands and has been found to be a transporter of some hydrophobic substances^3^. Originally, the main function of LCN2 was believed to be the capture and transport into the cytoplasm of iron ions, contributing to its bactericidal properties, among others. LCN2 is also categorized as a stress protein due to activation of iron-dependent defense systems following exposure to stress stimuli^4^. Recently, the oncogenic role of *LCN2* has been described in severe cancers, with higher *LCN2* expression in cancerous cells compared to non-cancerous cells^5^. Many studies have also identified a pro-neoplastic role for *LCN2* and related mechanisms^6^,^7^. However, controversies over its function remain. Some studies have shown that *LCN2* acts as a tumor suppressor gene in ovarian cancer, pancreatic cancer and colon cancer^8^,^9^,^10^. Studies investigating the role of LCN2 in CCA are still very limited.

The N-myc downstream regulated gene (NDRG) protein family comprises 4 members, NDRG1, NDRG2, NDRG3, and NDRG4^11^. NDRG proteins are widely expressed in human tissues, with *NDRG4* mainly expressed in the heart and brain^12^. *NDRG1* and *NDRG2* have been widely studied and identified as tumor suppressor genes in a variety of cancers^13^,^14^,^15^,^16^,^17^.

EMT is a process during which epithelial cells change towards a mesenchymal cell phenotype, playing a vital role in cancer cell metastasis. After EMT, cancer cells have increased motility and become more invasive. EMT also renders cancer cells more resistant to chemotherapy and surveillance of immune cells due to increased stem cell-like characteristics^18^,^19^,^20^.

MMPs are proteases that digest collagen, which is one of the main components of the extracellular matrix. Cancers with higher MMP expression tend to have higher invasiveness^21^,^22^.

Previously, our group has shown high *LCN2* expression in human CCA samples^23^. In the current study, we investigated the role of LCN2 in human CCA, including the effect of LCN2 on CCA cell growth and metastatic potential *in vitro*, and *in vivo* xenografted tumor growth. The relation between NDRGs and LCN2 in CCA cells were also studied for the first time. Furthermore, *LCN2* expression in human samples was studied to relate LCN2 levels to clinical characteristics and survival of patients with CCA. The level of LCN2 in bile in patients with CCA was measured for comparison with levels in gall stone patients. Overall, we aimed to provide a new therapeutic target and diagnostic marker for CCA.

## Results

### Characterization of *LCN2* mRNA expression in CCA cells

**Figure 1:**
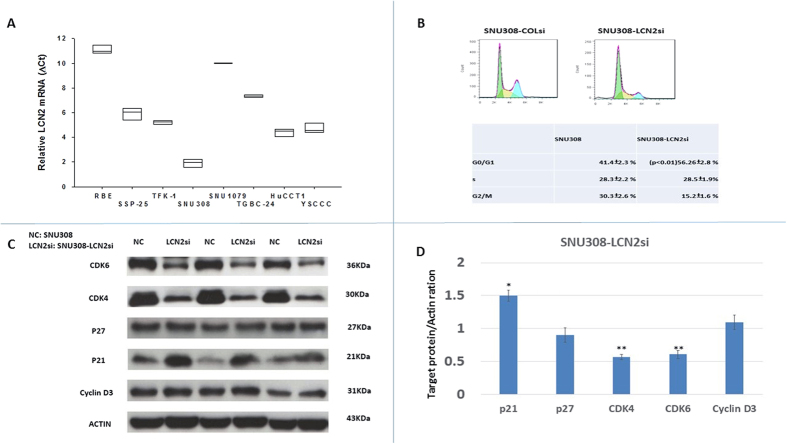
Expression of *LCN2* mRNA in 8 kinds of CCA cells and LCN2 effect on SNU308 cell cycle distribution. (**A**) *LCN2* mRNA expressions were measured in 8 kinds of CCA cells with RBE and SNU308 cells had the lowest and highest *LCN2* mRNA expressions, respectively. (**B**) Histogram of cell cycle distribution (upper panel) and quantitative result of cell cycle distribution (lower panel) of SNU308-COLsi and SNU308-LCN2si cells. (**C**) Western blots showing CDK4, CDK6, cyclin D3, p21, and p27 expression in SNU308-COLsi and SNU308-LCN2si cells. Experiments were done in triplicate and repeated at least three times. (**D**) Quantitative result of the western blot. Each value was a mean ± SD of three independent determinations. Data was presented as the intensity of protein bands of the target genes/β-actin relative to the control. Experiment was done at least three times (*P < 0.05).

*LCN2* expression was evaluated in 8 CCA cell lines: RBE, SSP-25, TFK-1, SNU308, SNU1079, TGBC-24, HUCCT1, and YSCCC. *LCN2* mRNA expression in each cell line was determined by RTqPCR. As shown in Fig. 1A, SNU308 cells had the highest level of *LCN2* mRNA expression, and expression was lowest in RBE cells.

### Effect of *LCN2* knockdown on CCA cell cycle progression and expressions of cell-cycle control related proteins

Previously we have shown that the doubling time of SNU308-LCN2si cells is increased as compared to SNU308-COLsi cells^23^, suggesting an oncogenic role for LCN2 in human CCA. Therefore, we further evaluated the effect of LCN2 on SNU308 cell cycle progression. As shown in Fig. 1B, SNU308-LCN2si cells had an increased number of cells in G0/G1 phase compared to SNU308-COLsi cells (56.26% and 41.44%, respectively), suggesting that knockdown of *LCN2* in SNU308 cells induces G0/G1 arrest, thus inhibiting cell growth. Further, to understand how LCN2 influences SNU308 cell cycle progression, the expression of: the cyclin dependent kinase inhibitors (CKIs) p21 and p27, cyclin dependent kinase (CDK) 4 and CDK6, and cyclin D3 were investigated. As shown in Fig. 1C,D, *LCN2* knockdown induced higher p21 expression and lower CDK4 and CDK6 expression in SNU308 cells with no obvious impact on p27 and cyclin D3 expression, initiating the G0/G1 arrest noted in Fig. 1B.

### Effect of *LCN2* knockdown on SNU308 metastatic potential

**Figure 2:**
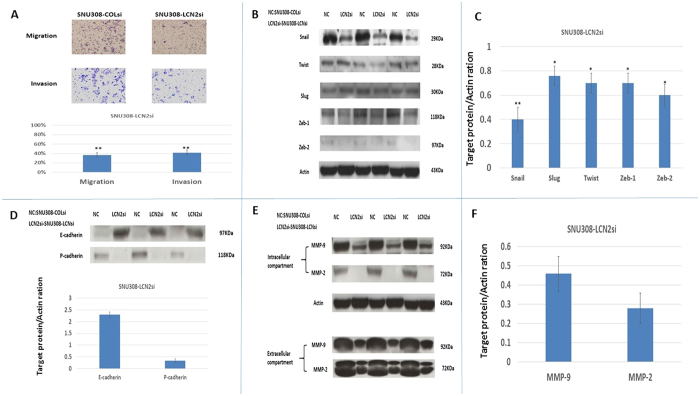
The effect of *LCN2* knockdown on SNU308 cell metastasis. (**A**) The migration and invasion ability of SNU308-COLsi and SNU308-LCN2si cells. Experiments were performed in triplicate and repeated at least three times. Data was presented as the ratio to the control (SU309-COLsi group) (**p < 0.01). (**B**) The western blot to show Snail, Slug, Twist, Zeb-1, and Zeb-2 expression in SNU308-COLsi and SNU308-LCN2si cells. (**C**) Quantitative result of the western blot shown in figure B. Each value was a mean ± SD of three independent determinations. Data was presented as the intensity of protein bands of the target genes/β-actin relative to the control. Experiment was done at least three times (*P < 0.05, **p < 0.01). (**D**) The western blot and quantitative analysis of E-cadherin and P-cadherin expression in SNU308-COLsi and SNU308-LCN2si cells. Each value was a mean ± SD of three independent determinations. Data was presented as the intensity of protein bands of the target genes/β-actin relative to the control. Experiment was done at least three times (**P < 0.01). (**E**) The western blot depicting intracellular and extracellular MMP-2 and MMP-9 expression in SNU308-COLsi and SNU308-LCN2si cells. (**F**) The quantitative analysis of the western blot shown in figure E. Each value was a mean ± SD of three independent determinations. Data was presented as the intensity of protein bands of the target genes/β-actin relative to the control. Experiment was done at least three times (**P < 0.01).

For evaluation of the role of *LCN2* in SNU308 cell metastatic potential, we then conducted invasion and migration assays. As shown in Fig. 2A, the population of SNU308-LCN2si cells had much fewer invading and migrating cells as compared to the population of SNU308-COLsi cells. This shows that *LCN2* knockdown attenuates metastatic potential. Since epithelial-mesenchymal transition (EMT) is a key step in cancer cell metastasis, we then investigated the effect of LCN2 on EMT-related transcription factors. Figure 2B,C demonstrate that Snail, Slug, Twist, Zeb1, and Zeb2 expression levels decreased in SNU308-LCN2si cells compared to SNU308-COLsi cells, implying that *LCN2* knockdown inhibited EMT. We further evaluated expression of E-cadherin and P-cadherin in SNU308 cells after *LCN2* knockdown. Figure 2D shows that E-cadherin was upregulated while P-cadherin was downregulated in SNU308-LCN2si cells. Intracellular and extracellular MMP-2 and MMP-9 expression were also decreased (Fig. 2E,F) after *LCN2* knockdown.

### Effect of *LCN2* overexpression on cell proliferation and metastatic potential in RBE cells

**Figure 3:**
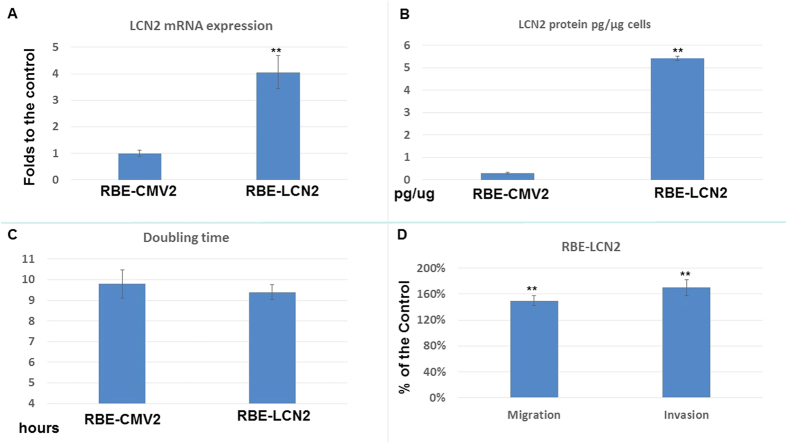
The effect of *LCN2* overexpression in RBE cell proliferation and metastasis. (**A**) *LCN2* mRNA expression in RBE-CMV2 and RBE-LCN2 cells. Each value was a mean ± SD of three independent determinations. Experiment was done at least three times (**P < 0.01). (**B**) ELISA assay to determine the secreted LCN2 amounts of RBE-CMV2 and RBE-LCN2 cells. Each value was a mean ± SD of three independent determinations. Experiment was done at least three times (**P < 0.01). (**C**) The doubling time of RBE-CMV2 and RBE-LCN2 cells. (**D**) The migration and invasion ability of RBE-LCN2 cells as compared to RBE-CMV2 cells. Each value was a mean ± SD of three independent determinations. Experiment was done at least three times. Data was presented as the ratio to the control (RBE-CMV2 group) (**P < 0.01).

*LCN2* overexpression was induced in RBE cells (Fig. 3A) to evaluate its effect on RBE cell proliferation, invasion, and migration. RBE-LCN2 cells exhibited higher *LCN2* mRNA expression and LCN2 secretion than RBE-CMV2 cells (Mock transfection of *LCN2*) (Fig. 3A,B). Figure 3C shows that RBE-LCN2 cells had a similar doubling time to RBE-CMV2 cells (The calculation of double time was described previously ref. 23). Figure 3D demonstrate that migration and invasion were increased to 155 ± 5% and 165 ± 7% respectively in RBE-LCN2 cells as compared to RBE-CMV2 cells.

### Effect of *LCN2* knockdown or overexpression on *NDRG1* and *NDRG2* expression in CCA cells

**Figure 4:**
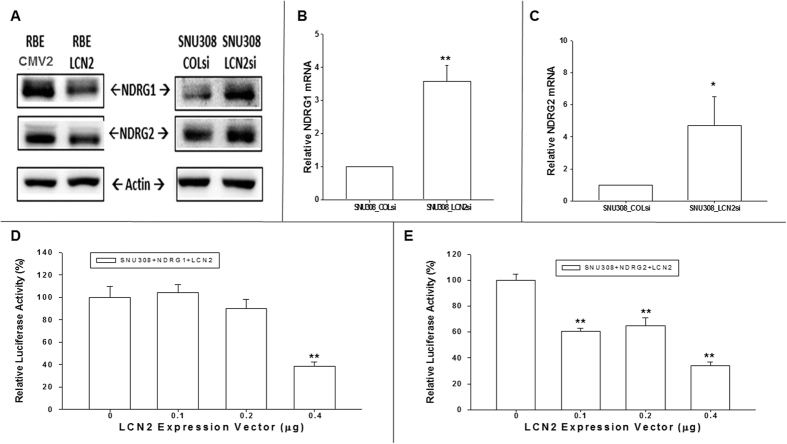
Evaluation *LCN2* effect on NDRG1 and NDRG2 expressions in CCA cells. (**A**) The western blot to show the downregulation or upregulation of NDRG1 and NDRG2 as *LCN2* overexpression in RBE cells or knockdown in SNU308 cells. (**B**) Relative NDRG1 mRNA expression of SNU308-LCN2si cells to SNU308-COLsi cells. Each value was a mean ± SD of three independent determinations. Experiment was done at least three times (**P < 0.01). (**C**) Relative NDRG2 mRNA expression of SNU308-LCN2si cells to SNU308-COLsi cells. Each value was a mean ± SD of three independent determinations. Experiment was done at least three times (**P < 0.01). (**D**) The activity of NDRG1 reporter in SNU308 cells as treated with indicated concentrations of LCN2 expression vectors. Each value was a mean ± SD of three independent determinations. Experiment was done at least three times (**P < 0.01). (**E**) The activity of NDRG2 reporter in SNU308 cells as treated with indicated concentrations of LCN2 expression vectors. Each value was a mean ± SD of three independent determinations. Experiment was done at least three times (**P < 0.01).

The *NDRG* gene family is widely regarded as comprising several tumor suppressor genes. *NDRG1* and *NDRG2* expression was evaluated following *LCN2* overexpression or knockdown. Figure 4A reveals that the expression of *NDRG1* and *NDRG2* was increased after *LCN2* knockdown in SNU308 cells but decreased after *LCN2* overexpression in RBE cells, as determined by western blot. This result is supported by the higher expression of *NDRG1* and *NDRG2* mRNA in SNU308-LCN2si cells as compared to SNU308-COLsi cells (Fig. 4B,C). The reporter assay confirms this, as transiently transfecting SNU308 cells with an *NDRG1* reporter using an *LCN2* expression vector decreased reporter activity (Fig. 4D). Similar results were observed in the *NDRG2* reporter assay (Fig. 4E). Overall, we concluded that NDRG1 and NDRG2 are downstream of LCN2 in CCA cells.

### Effect of *LCN2* knockdown on SNU308 cell growth *in vivo*

**Figure 5:**
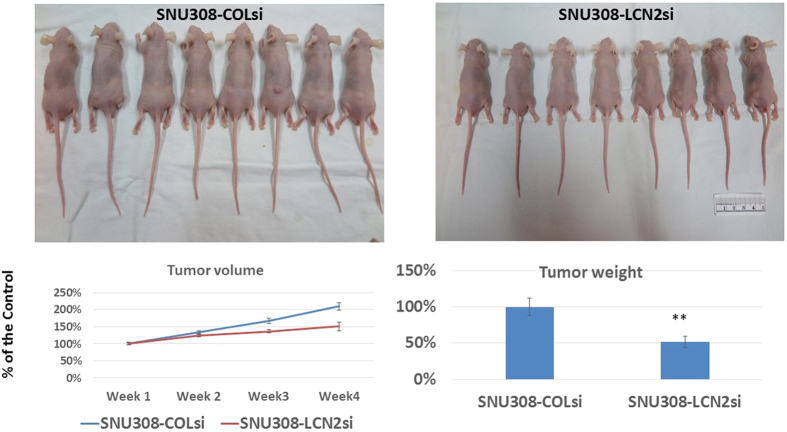
*LCN2* knockdown repressed SNU308 cell growth *in vivo*. SNU308-COLsi and SNU308-LCN2si cells were xenografted into the nude mice ( = 8 for each group). Four weeks later, the tumors were harvested and weighted. Our result clearly shows that LCN2 knockdown significantly inhibited SNU308 cell growth *in vivo* (**P < 0.01).

To investigate the effect of *LCN2* knockdown on SNU308 cell growth *in vivo*, tumors comprised of either SNU308-LCN2si cells or SNU308-COLsi cells were xenografted into nude mice. 4 weeks later, the xenografted tumors were harvested and weighed. Figure 5 shows that tumors from the SNU308-LCN2si cell group had a much lower tumor weight and smaller tumor volume than that in the SNU308-COLsi cell group, indicating that *LCN2* knockdown was able to inhibit CCA cell growth *in vivo*.

### Evaluation of LCN2 concentration in bile from CCA or gallstone patients

**Figure 6:**
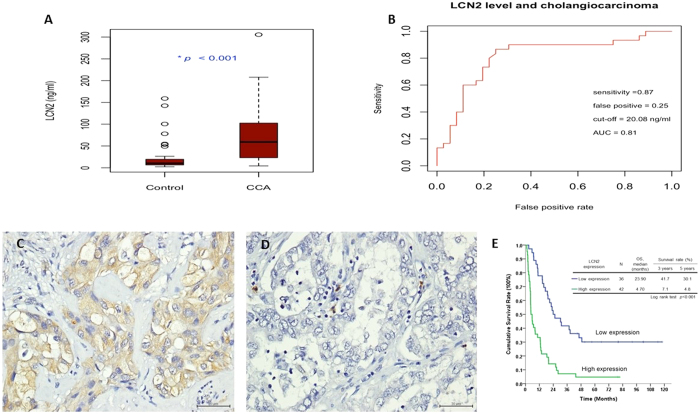
CCA patients had higher bile LCN2 level than patients with gall stones and *LCN2* expression was negatively correlated with survival of CCA patients. (**A**) The bile LCN2 level for CCA patients was statically higher than that for patients with gall stones (P < 0.001). (**B**) The AUC was 0.81 as analyzing bile LCN2 level by ROC curve. The optimal cutoff value of LCN2 that had a largest Youden index was 20.08 ng/ml, which had a sensitivity of 0.87 and false positive rate of 0.25 to differentiate CCA patients to non-CCA patients. (**C**,**D**) Immunohistochemical staining of human CCA specimen with different intensity scores for LCN2 expression (high and low, respectively; X400, Scale bar = 50 μm). (**E**) Kaplan–Meier plot of overall survival in CCA patients based on LCN2 expression levels. The high LCN2 expression group had poorer overall survival (*P* < 0.001).

The median LCN2 levels in the bile for 30 patients with CCA and 36 gallstone patients were 59.26 ng/ml and 10.19 ng/ml, respectively. The LCN2 level in patients with CCA was statistically higher than that in gallstone patients (P < 0.001, Fig. 6A). The area under the ROC curve was 0.81 with an optimal cutoff value, which had the largest Youden index, of 20.08 ng/ml. The test had a sensitivity of 0.87 and a false positive rate of 0.25 (Fig. 6B).

### Relationship between LCN2 expression and clinicopathological characteristics of patients with CCA

*LCN2* was diffusely expressed in the cytoplasm of CCA cells with 42 (53.8%) patients graded as exhibiting high expression (based on H-scores ≥ 40) (Fig. 6C,D). Higher *LCN2* expression was associated with lower albumin level and a higher rate of positive surgical margin in patients with CCA (Supplemental Table 1). After univariate and subsequent multivariate analysis, only *LCN2* expression was found to be negatively associated with overall survival (OS) of patients with CCA (p < 0.001, relative risk, 3.615 (1.721–7.592)) (Supplemental Table 2 and Fig. 6E).

## Discussion

*LCN2* is widely deemed as an oncogene in a variety of cancers despite some enduring controversy^6^, but studies regarding LCN2 anti-cancer mechanisms remain limited. Moreover, the fact that the findings of these studies are sometimes contradictory indicates that LCN2 may work in a highly cell- or tissue- specific manner. Our results demonstrate that *LCN2* acts as an oncogene in human CCA, as *LCN2* expression was positively correlated with CCA cell growth and metastatic potential and negatively associated with CCA patient survival. We investigated the mechanisms whereby LCN2 influences CCA cell growth and metastasis. Furthermore, the bile of patients with CCA exhibited a much higher LCN2 concentration compared to individuals without CCA. Taken together, our data suggest that *LCN2* could be a promising therapeutic target and diagnostic marker for human CCA.

Cell cycle progression in normal cells is under strict control to maintain homeostasis. However, due to constitutively high mitogenic signaling in cancer, the cell cycle of cancer cells is usually dysregulated, leading to uncontrolled proliferation. Thus, targeting cell cycle progression is an effective way to curb cancer cell growth^24^. To evaluate the effect of LCN2 on CCA cell cycle progression, 8 CCA cell lines were analyzed for *LCN2* mRNA expression, with SNU308 showing the highest *LCN2* expression (Fig. 1A). We therefore knocked down *LCN2* in SNU308 cells and analyzed the distribution of cells at each stage of the cell cycle within the population. Figure 1B reveals that more SNU308-LCN2si cells were in G0/G1 phase compared to SNU308-COlsi cells, indicating that *LCN2* knockdown can induce cell cycle arrest at G0/G1.

To initiate cell division, cells must pass a restriction point located in mid to late G1 phase, for which release of E2F-1 from its inhibitor, retinoblastoma protein (RB) is necessary^25^. This requires RB to be phosphorylated, involving specific CDKs and cyclins, and is negatively controlled by specific CKIs. Among others, CDK4, CDK6, and cyclin D3 are responsible for G1/S transition. Figure 1C,D demonstrate that CDK4 and CDK6 expression was decreased in SNU308-LCN2si cells, while no obvious change in expression of cyclin D3 occurred. Two CKI initiators of G1/S transition, p21 and p27, were also examined. As shown in Fig. 1C,D, *LCN2* knockdown increased p21 expression with no significant impact on p27. Taken together, we concluded that *LCN2* knockdown in SNU308 cells can retard cell cycle progression at G0/G1 phase by downregulation of CDK4 and CDK6 and upregulation of p21, leading to the inhibition of cell proliferation noted in our previous study^23^. Following xenograft of SNU308-LCNsi or SNU308-COLsi tumors into nude mice, tumor growth from SNU308-LCNsi cells was much slower than from SNU308-COLsi cells (Fig. 5). Collectively, our results suggest that *LCN2* knockdown could repress SNU308 cell growth *in vitro* and *in vivo*.

It has been reported that EMT is positively associated with cancer progression, resulting in poor prognosis^26^. The regulation of EMT process is well orchestrated. Among others, Snail, Slug, Twist, Zeb1, and Zeb2 are transcriptional factors responsible for EMT induction^27^. Figure 2B,C demonstrate that all five transcriptional factors were downregulated following *LCN2* knockdown in SNU308 cells, indicating that *LCN2* knockdown could inhibit EMT progress in CCA cells, attenuating the metastatic potential noted in Fig. 2A. Another hallmark of EMT is the downregulation of E-cadherin^28^. To initiate metastasis, cancer cells must detach from the primary tumor first. E-cadherin, a transmembrane glycoprotein, plays a vital role in calcium-dependent cell-cell adhesion^29^,^30^ and its expression has been negatively associated with cancer invasiveness and prognosis^31^,^32^. Figure 2D shows that SNU308-LCNsi cells expressed E-cadherin more highly than SNU308-COLsi cells, further supporting the finding that the EMT process is attenuated by *LCN2* knockdown in SNU308 cells. The downregulation of P-cadherin, which is another marker of mesenchymal cells and linked with CCA cell migration^33^, in *LCN2* knockdown SNU308 cells also demonstrated EMT process was repressed in SNU308-LCNsi cells (Fig. 2D).

Previously, LCN2 has been shown to form an LCN2/MMP complex that protects MMP-9 from degradation, thus increasing MMP-9 activity^34^. In CCA, *LCN2* knockdown in CCA cells had been shown to repress invasion through reduction of LCN2/MMP-9 complex formation^35^. Our result indicates that knockdown of *LCN2* in CCA cells could decrease both intracellular and extracellular MMP-2 and MMP-9 expression, reducing cell invasiveness (Fig. 2D,E).

The effect of LCN2 on CCA cells was further demonstrated through overexpression in RBE cells (Fig. 3). It is obvious that RBE-LCN2 cells exhibited higher migration and invasion abilities than RBE-CMV2 cells, although cell proliferation was similar in both cell types (Fig. 3B–D).

NDRG1 has been reported to induce differentiation, cell cycle arrest, and cell growth inhibition in a variety of cancers^36^. Similar to NDRG1, many studies have implied an anti-cancer role for NDRG2^37^. Since *NDRG1* and *NDRG2* belong to the N-Myc downstream-regulated gene family, their expression is modulated by N-myc. Besides N-myc, *NDRG1* and *NDRG2* have also been reported to be regulated by p53, HIF-1α, and PTEN, among others^14^,^38^,^39^,^40^,^41^. As shown in Fig. 4, as we knocked down or overexpressed *LCN2* in CCA cells, NDRG1 and NDRG2 protein expression was increased or decreased, respectively (Fig. 4A). The RTqPCR and reporter assay also confirmed that both *NDRG1* and *NDRG2* expression was regulated by LCN2 (Fig. 4B–E). We showed for the first time that *NDRG1* and *NDRG2* are downstream targets of LCN2 in CCA cells.

*LCN2* has been applied as a tumor marker in bladder cancer and has been found to be higher in urine of patients’ diagnosed with non-papillary bladder cancer as compared to papillary bladder cancer^42^. To evaluate the clinical relevance of *LCN2* expression in CCA, bile from CCA and gall stone patients was collected for measurement of LCN2 concentration. Medium concentration of 59.26 ng/ml or 10.19 ng/ml were obtained for CCA or gall stone patients, respectively, with the optimal cutoff value to differentiate patients with CCA from those without CCA being 20.08 ng/ml (sensitivity of 0.87 and false positive rate of 0.25). Our result suggests that LCN2 concentration in bile could be a valued diagnostic marker for human CCA. Other report also demonstrated the similar result although the cutoff value was much higher than our current finding, which may be due to they recruited both malignant or benign biliary obstruction patients for comparison^43^.

*LCN2* has been shown to be able to render cancer cells more resistant to chemotherapy and target therapy^44^ and has been adversely associated with cancer patients’ survival^45^,^46^. In our current study, higher *LCN2* expression was linked with poor OS of patients with CCA (Fig. 6C–E), further justifying the development of LCN2-targeting therapies in CCA treatment.

Our current study indicates that *LCN2* could be a promising target for CCA treatment, since it acts as an oncogene in CCA cells *in vitro* and *in vivo,* and higher *LCN2* expression is linked to poor OS of patients with CCA. In conjunction with the finding that bile LCN2 concentration was higher in patients with CCA, we conclude that in addition to being a potential therapeutic target, bile LCN2 concentration is a good diagnostic marker for CCA.

## Materials and Methods

### Cell culture

SNU308 and RBE cells, human CCA cell lines, were purchased from the Korean Cell Line Bank (KCLB: 28 Yongon-dong, Chongno-gu, Seoul 110–744, Korea). The cells were tested using the PromegaGenePrint 10 System and mRNA was analyzed using ABI PRISM 3730 GENETIC ANALYZER and GeneMapper Software V3.7.

### Knockdown of *LCN2* in SNU308 cells

SNU308 cells were transducted with control small hairpin RNA lentiviral particles (Sc-10808-V, Santa Cruz Biotechnology) according to the manufacturer’s instructions. Two days after transduction, the cells (SNU-COLsi and SNU-NGALsi) were selected by incubation with 10 μg/ml puromycin dihydrochloride for another 3 generations. The detailed procedures were described previously^23^.

### *LCN2* expression vector constructs and stable transfection

The expression vector containing coding region of LCN2 (HG10222-M-Y) was purchased from Sino Biological Inc (Beijing, China). The expression vector was introduced into REB cells by electroporation. The LCN2-transfected cells (REB-LCN2) were selected with hygromycin B (50 μg/ml; Invitrogen, Carlsbad, CA) for at least 4 generations. The mock-transfection (REB-CMV2) cells were transfected with pCMV2HA expression vector (Sino Biological Inc) and were selected as REB-LCN2 cells.

### Cell cycle analysis

Cells were serum starved for the 24 hours preceding analysis as previously described^47^,^48^. The cells were fixed in ice-cold 75% ethanol. The fixed cells were stained by propidium iodide (PI) buffer containing 100 mM sodium citrate, 0.1% Triton X-100, 0.2 mg/ml RNase, and 50 *μ*g/ml PI at 4 °C for 1 h. Cell cycle analysis was performed using a FACSCalibur cytometer and CellQuest Pro software (BD Biosciences, San Jose, CA).

### Matrigel invasion assay

The matrigel invasion assay was conducted as previously described^49^. 48 hours afterwards, the invading cells were fixed with 4% paraformaldehyde in 1 × PBS, stained, digitally photographed and counted under the microscope (IX71, Olympus, Tokyo, Japan). Experiments were performed in triplicate and repeated at least three times.

### Trans-well filter migration assay

The migration assay was conducted as previously described^50^. 24 hours afterwards, the migrating cells were stained and counted under four random high-power microscopic fields (HPF; 100X) per filter. The experiments were performed in triplicate and repeated at least three times.

### Real-time reverse transcription-polymerase chain reaction (RT-qPCR)

Total RNA Isolation kit (Promega, Madison WI, USA) was applied to extrat total RNA. For each sample, cDNA was generated from 2 μg of RNA using Superscript RNAase H- (Invitrogen,Carlsbad, CA, USA) with random hexamer primers. qPCR was performed using an ABI StepOne Plus Real-Time PCR system (Applied Biosystems, Foster City, CA, USA). FAM dye-labeled TaqMan MGB probes for human *NDRG1* (Hs00608387_m1), *NDRG2* (Hs01045115_m1), *LCN2* (Hs00194353_m1) and β-actin (Hs01060665_g1) were purchased from Applied Biosystems.

### Western blotting

Western blots were performed as described previously^47^. Cells were washed once with PBS and lyzed in the lysis buffer containing 50 mM Tris-Cl, 50 mM β-glycerolphosphate, 50 mM NaCl, 1 mM Na_3_Vo_4_, 1 mM EDTA, 1 mM EGTA, 1% NP40, and freshly adding 1 mM DTT, 1 mM PMSF, 2 μg/ml Aprodenin, 2 μg/ml Leupeptin, and 2 μg/ml Pepstatin right before lysis. The cell lysates were then rotated for 30 minutes and centrifuged at 13,000 g for 30 minutes at 4 °C and the supernatant was collected. The protein concentration of the cell lysate was determined by using BCA reagent (Thermo Scientific Pierce) with BSA as standards. The antibodies used in this experiment are listed in the Supplementary data.

### Reporter assay for NDRG1 and NDRG2

The *NDRG1* (−4714 to +46) reporter vectors were constructed as described previously^51^. A 5.2 kbp DNA fragment was subtracted from a BAC clone (PR11-998D10; Invitrogen) and cloned into the pGEM-3 vector (Promega BioScience) with the *Kpn I* cutting site. The DNA fragment containing the enhancer/promoter of the *NDRG2* gene (−4253 to −1) was synthesized with primers (Sp6 and 5′-AAGCTTCTATAAATAGAGGGCGATCGC-3′) by PCR using pGEM-3NDRG2 as target DNA. This DNA fragment was cloned into the pGL3-Basic vector (Promega BioScience) vector at the *Hind III* cutting sites. Cells were then transiently transfected and luciferase activity was determined in relative light units (RLU) as described previously^17^.

### Tumor xenografts

This study was approved by the Chang Gung University Animal Research Committee (Permit Number: 2014022601) and carried out in accordance with the guidelines. Equal volumes of tumor cells and matrigel were mixed (total 100 μl, containing 5 × 10^6^ cells) and injected into the dorsal region of nude mice (BALB/cAnN-Foxn1, 4 weeks old). The weight of the xenografts was measured after 4 weeks.

### Patient demographics for IHC analysis and bile collection

Patients with CCA, who underwent hepatectomy between 1989 and 2006 at the Department of Surgery at Chang Gung Memorial Hospital were enrolled (N = 78). The study was approved by the local institutional review board of Chang Gung Memorial Hospital (clinical study numbers 99-2886B, 99-3810B and 102-5813B) and informed consents were obtained for all subjects. In 2015, another cohort of 66 patients, undergoing treatment for either gallstones or CCA were enrolled in the study. For gallstone patients (N = 36), bile samples were aspirated from gallbladder immediately after surgery. For patients with CCA (N = 30), bile samples were collected from the percutaneous transhepatic-cholangio-drainage tube.

### LCN2 immunohistochemistry and LCN2 level in bile

LCN2 expression levels of samples from the aforementioned 78 patients with CCA were examined by immunohistochemistry (IHC) as previously described^23^. For the assessment of IHC, H scores were calculated as the percentage of positive staining (0–100)× the corresponding staining intensity (0–3). Specimens with H-scores of <40 or ≥40 were classified as having low or high expression respectively. The level of LCN2 in bile was measured using a commercially available ELISA kit (Catalog #: DLCN20; R&D Systems, Inc., Minneapolis, MN).

### Statistical analyses

All data were presented as the mean + standard deviation (SD). Differences between the experimental and control groups were calculated using the student’s *t*-test. Differences in tumor weights between the experimental and control animals, and LCN2 levels in bile between the two groups of patients were compared by the Mann-Whitney U test. The cut-off value of LCN2 level was determined by receiver operating characteristic (ROC) curve analysis^52^. Overall survival rate was evaluated using the Kaplan–Meier method. Several clinicopathological variables were considered for the initial single-variable analysis, which was performed with the log-rank test. The Cox proportional hazards model was applied for multivariate regression. A value of *p* ≤ 0.05 derived from a 2-tailed test was considered statistically significant.

## Additional Information

**How to cite this article**: Chiang, K.-C. *et al*. Lipocalin 2 (LCN2) is a promising target for cholangiocarcinoma treatment and bile LCN2 level is a potential cholangiocarcinoma diagnostic marker. *Sci. Rep.* **6**, 36138; doi: 10.1038/srep36138 (2016).

**Publisher’s note:** Springer Nature remains neutral with regard to jurisdictional claims in published maps and institutional affiliations.

## Supplementary Material

Supplementary Information
