# Supplementary material for: Lipocalin 2 (LCN2) is a promising target for cholangiocarcinoma treatment and bile LCN2 level is a potential cholangiocarcinoma diagnostic marker
Source: Sci Rep. 2016 Oct 26;6:36138. doi: 10.1038/srep36138 (PMC5080596; doi:10.1038/srep36138)
Supplement: Supplementary Information [file srep36138-s1.doc]

**Lipocalin 2 (LCN2) is a promising target for cholangiocarcinoma treatment and bile LCN2 level is a potential cholangiocarcinoma diagnostic marker**

Kun-Chun Chiang^1,2*^,Ta-Sen Yeh^3*^, Ren-Chin Wu^4^, Jong-Hwei S. Pang^5^, Chi-Tung Cheng^3^, Shang–Yu Wang^3^, Horng-Heng Juang^6,7#^,Chun-Nan Yeh^3#^

^1^General Surgery Department, Chang Gung Memorial Hospital, Keelung, Taiwan, R.O.C, Chang Gung University

^2^ Director of Zebrafish center of Keelung Chang Gung Memorial Hospital, Taiwan, R.O.C

^3^General Surgery Department and Liver research center, Chang Gung Memorial Hospital, Kwei-Shan, Taoyuan, Taiwan, R.O.C, Chang Gung University

^4^Department of Pathology and Liver research center, Chang Gung Memorial Hospital, Kwei-Shan, Taoyuan, Taiwan, R.O.C, Chang Gung University

^5^Graduate Institute of Clinical Medical Sciences, College of Medicine, Chang Gung University, Kwei-Shan, Taoyuan, Taiwan, R.O.C.

^6^Department of Anatomy, College of Medicine, Chang Gung University, Kwei-Shan Taoyuan, Taiwan, 333, R.O.C.

^7^Department of Urology, Chang Gung Memorial Hospital, Kwei-Shan, Tao-Yuan, Taiwan, ROC

| Table 1 Clinicopathological features between LCN2 high expression and low expression of cholangiocarcinoma patients | | | |
| --- | --- | --- | --- |
|  | LCN2  low expression  (n=36) | LCN2  high expression (n=42) | *p* |
| Age (years) | 58.83±12.28 | 60.54±11.81 |  |
| Gender |  |  | 0.572 |
| Male | 14(42.4%) | 19(57.6%) |  |
| Female | 22(48.9%) | 23(51.1%) |  |
| Symptom |  |  | 0.358 |
| Negative | 7(58.3%) | 5(41.7%) |  |
| Positive | 29(43.9%) | 37(56.1%) |  |
| AST (IU/l) |  |  | 0.915 |
| ≦34 | 18(46.2%) | 21(53.8%) |  |
| >34 | 18(47.4%) | 20(52.6%) |  |
| ALT (U/L) |  |  | 0.941 |
| ≦36 | 19(47.5%) | 21(52.5%) |  |
| >36 | 15(48.4%) | 16(51.6%) |  |
| ALP (U/L) |  |  | 0.101 |
| ≦94 | 14(60.9%) | 9(39.1%) |  |
| >94 | 21(40.4%) | 31(59.6%) |  |
| Bilirubin (total) (mg/dl) |  |  | 0.133 |
| ≦1.3 | 27(42.2%) | 37(57.8%) |  |
| >1.3 | 9(64.3%) | 5(35.7%) |  |
| Albumin (g/dl) |  |  | 0.048^*^ |
| ≦3.5 | 7(31.8%) | 15(68.2%) |  |
| >3.5 | 28(57.1%) | 21(42.9%) |  |
| Serum CEA (ng/ml) |  |  | 0.214 |
| ≦5 | 13(52.0%) | 12(48.0%) |  |
| >5 | 11(35.5%) | 20(64.5%) |  |
| Size (cm) |  |  | 0.618 |
| ≦5 | 16(50.0%) | 16(50.0%) |  |
| >5 | 19(44.2%) | 24(55.8%) |  |
| Lymph node |  |  | 0.411 |
| Negative | 24(51.1%) | 23(48.9%) |  |
| Positive | 12(41.4%) | 17(58.6%) |  |
| Differentiated |  |  | 0.682^b^ |
| Well | 0(00.0%) | 2(100.0%) |  |
| Moderate | 18(43.9%) | 23(56.1%) |  |
| Poorly | 17(51.5%) | 16(48.5%) |  |
| other | 1(50.0%) | 1(50.0%) |  |
| Margin |  |  | 0.016^*^ |
| Negative | 31(54.4%) | 26(45.6%) |  |
| positive | 5(23.8%) | 16(76.2%) |  |
| Hepatitis B |  |  | 0.663 |
| Negative | 25(50.0%) | 25(50.0%) |  |
| positive | 9(56.3%) | 7(43.8%) |  |
| Hepatitis C |  |  | 0.540 |
| Negative | 29(50.9%) | 28(49.1%) |  |
| positive | 5(55.6%) | 4(44.4%) |  |
| Post Chemotherapy |  |  | 0.137 |
| Without | 12(36.4%) | 21(63.6%) |  |
| With | 24(53.3%) | 21(46.7%) |  |
| Post Radiotherapy |  |  | 0.472^b^ |
| Without | 32(47.1%) | 36(52.9%) |  |
| With | 4(40.0%) | 6(60.0%) |  |

| **Table 2. Univariate and Multivariate Analysis of Factors Influencing the Overall Survival of the 78 MF-CCA Patients** | | | | | |
| --- | --- | --- | --- | --- | --- |
|  | Median  Survival Time (months) | 95% CI of Median | Univariate P | Relative Risk  (95% confidence interval) | Multivariate P |
| Gender |  |  | **0.719** |  |  |
| Male (n=33) | 14.70 | 7.89-21.50 |  |  |  |
| Female (n=45) | 10.82 | 5.85-15.79 |  |  |  |
| Age |  |  | **0.505** |  |  |
| ≦60 (n=38) | 12.89 | 4.45-21.33 |  |  |  |
| >60 (n=40) | 12.99 | 7.23-18.74 |  |  |  |
| Symptoms |  |  | **0.006** | **3.006(0.666-13.570)** | **0.152** |
| Negative (n=12) | 37.71 | 4.78-70.63 |  |  |  |
| Positive (n=66) | 10.45 | 5.84-15.07 |  |  |  |
| AST (IU/l) |  |  | **0.197** |  |  |
| **≦**34 (n=39) | 13.32 | 9.53-17.10 |  |  |  |
| >34 (n=38) | 10.72 | 2.28-19.16 |  |  |  |
| ALT (IU/l) |  |  | **0.625** |  |  |
| **≦**36 (n=40) | 12.99 | 6.41-19.56 |  |  |  |
| >36 (n=31) | 14.70 | 7.81-21.58 |  |  |  |
| ALP (IU/L) |  |  | **0.009** | **1.771(0.761-4.120)** | **0.185** |
| **≦**94 (n=23) | 23.90 | 11.65-36.15 |  |  |  |
| >94 (n=52) | 9.11 | 4.92-13.29 |  |  |  |
| Bil (total) (mg/dl) |  |  | **0.581** |  |  |
| **≦**1.3 (n=64) | 12.99 | 6.41-19.56 |  |  |  |
| >1.3 (n=14) | 10.72 | 0.00-22.23 |  |  |  |
| Albumin (g/dl) |  |  | **0.063** |  |  |
| **≦**3.5 (n=22) | 4.70 | 3.11-6.29 |  |  |  |
| >3.5 (n=49) | 19.04 | 13.31-24.76 |  |  |  |
| Serum CEA (ng/dl) |  |  | **0.043** | **0.759(0.347-1.659)** | **0.489** |
| **≦**5 (n=25) | 18.51 | 2.09-34.93 |  |  |  |
| >5 (n=31) | 10.29 | 4.05-16.53 |  |  |  |
| Margin |  |  | **<0.001** | **1.537(0.733-3.224)** | **0.255** |
| Negative (n=54) | 19.43 | 14.97-23.89 |  |  |  |
| Positive (n=24) | 4.41 | 2.43-6.38 |  |  |  |
| Size |  |  | **0.006** | **1.468(0.708-3.044)** | **0.302** |
| **≦**5cm (n=32) | 19.99 | 13.75-26.23 |  |  |  |
| >5cm (n=43) | 9.11 | 1.97-16.25 |  |  |  |
| Lymph node |  |  | **0.063** |  |  |
| Negative (n=47) | 19.89 | 16.71-23.07 |  |  |  |
| Positive (n=29) | 10.45 | 0.00-22.71 |  |  |  |
| Histology |  |  | **0.207** |  |  |
| Well (n=2) | 2.73 | NA |  |  |  |
| Moderate (n=41) | 13.84 | 7.45-20.23 |  |  |  |
| Poor (n=33) | 12.99 | 5.25-20.72 |  |  |  |
| Others(n=2) | 4.37 | NA |  |  |  |
| LCN2 |  |  | **<0.001** | **3.615(1.721-7.592)** | **0.001** |
| Low expression (n=36) | 23.90 | 12.06-35.74 |  |  |  |
| High expression (n=42) | 4.70 | 2.93-6.48 |  |  |  |
| Hepatitis B |  |  | **0.243** |  |  |
| Negative (n=50) | 12.89 | 8.29-17.48 |  |  |  |
| Positive (n=16) | 19.99 | 10.01-29.91 |  |  |  |
| Hepatitis C |  |  | **0.904** |  |  |
| Negative (n=57) | 14.53 | 9.84-19.22 |  |  |  |
| Positive (n=9) | 12.25 | 0.0-42.52 |  |  |  |
| Post-op Chemotherapy |  |  | **0.937** |  |  |
| Without (n=5) | 5.65 | 2.47-8.84 |  |  |  |
| With (n=45) | 14.70 | 9.64-19.75 |  |  |  |
| Post-op Radiotherapy |  |  | **0.071** |  |  |
| Without (n=68) | 13.32 | 8.27-18.36 |  |  |  |
| With (n=10) | 6.97 | 5.29-8.65 |  |  |  |
| AST: aspartate aminotransferase; ALT: alanine amionotransferase; ALP: alkaline phosphatase; CEA: carcinoembryonal antigen; CA 19-9: carbohydrate antigen; IU: international unit; op: operation | | | | | |

The antibodies used in this study:

The primary antibodies used in this study were monoclonal antibodies against CDK4 (cell signal, #2906,), CDK6 (cell signal, #3136), p21 (#2946, Cell Signal), p27 (Cell Signal, #3698), , cyclin D3 (cell signal, #2936), LCN2 (#PAB9543, Abnova Corporation, Taipei, Taiwan) E-cadherin (cell signal, #3195), P-cadherin (cell signal, #2189), Zeb1 ( TA802313, OriGene Technologies, Inc, Rockville, MD, USA) Zeb 2 (TA802113, OriGene Technologies, Inc, Rockville, MD, USA), Snail ( PA5-23472, Thermo Fisher Scientific, Waltham, MA, USA), Slug ( cell signal, #9585), Twist (sc-15393, Santa Cruz Biotechnology, Texas, USA), Zeb-1 (sigma, HPA027524), Zeb-2(sigma, HPA003456), NDRG1 (42-6200, Invitrogen), NDRG2 (ab169775, Abcam).
